# Enhanced tribological properties of diesel-based engine oil through synergistic MoS_2_-graphene nanohybrid additive

**DOI:** 10.1038/s41598-023-43260-1

**Published:** 2023-10-13

**Authors:** Thachnatharen Nagarajan, Nanthini Sridewi, Weng Pin Wong, Rashmi Walvekar, Mohammad Khalid

**Affiliations:** 1 Faculty of Defence Science and Technology, National Defence University of Malaysia, Kuala Lumpur, Malaysia; 2 Sunway Centre for Electrochemical Energy and Sustainable Technology (SCEEST), School of Engineering and Technology, Sunway University, No. 5 Jalan Universiti, Bandar Sunway, 47500 Petaling Jaya, Selangor Malaysia; 3 Faculty of Innovation and Technology, School of Engineering, Chemical Engineering Programme, Taylor’s University Malaysia, No.1 Jalan Taylor’s, 47500 Subang Jaya, Selangor Malaysia; 4 Liveable Urban Communities Impact Lab, Taylor’s University Malaysia, No.1 Jalan Taylor’s, 47500 Subang Jaya, Selangor Malaysia; 5 Manipal Institute of Technology, Manipal Academy of Higher Education, Manipal, Karnataka 576104 India; 6 Uttaranchal University, Dehradun, Uttarakhand 248007 India

**Keywords:** Graphene, Synthesis and processing, Graphene

## Abstract

This research explores the potential of microwave-synthesized MoS_2_-graphene nanohybrid as additives to enhance the tribological properties of diesel-based engine oil. The synthesis method offers significant advantages, reducing both synthesis time and energy consumption by 90–98% compared to conventional approaches. The synthesized nanohybrids are characterized through FESEM, EDX, XRD, and Raman spectroscopy to understand their morphology and functional group interactions. These nanohybrids are incorporated into 20W40 engine oil following synthesis, and a comprehensive assessment of their properties is conducted. This evaluation covers critical parameters like viscosity index, stability, volatility, as well as tribological properties, oxidation resistance, and thermal conductivity of the oil-nanohybrid system. Results demonstrate that adding just 0.05 wt% of MoS_2_-graphene nanohybrid leads to a remarkable 58.82% reduction in friction coefficient and a significant 36.26% decrease in the average wear scar diameter. Additionally, oxidation resistance improves by 19.21%, while thermal conductivity increases notably by 19.83% (at 100 °C). The study demonstrates the synergistic effects of these nanohybrids in reducing friction and wear, enhancing oxidation resistance, and improving thermal conductivity. In conclusion, this research highlights the potential of microwave-synthesized MoS_2_-graphene nanohybrid as promising tribological additives for diesel engine oils. Their successful integration could significantly enhance the performance and durability of critical mechanical components in diesel engines, representing a significant advancement in lubrication technology.

## Introduction

Lubrication of engine components is essential for ensuring efficient operation, minimizing wear and tear, and extending the engine’s lifespan. One of the latest advancements in engine lubrication is the use of nanolubricants, which are designed to enhance the performance of conventional lubricants by reducing friction and wear at the nanoscale level^1^. Nanolubricants are typically composed of a base lubricant, such as mineral or synthetic oil, and nanoparticles that are added to the base lubricant to improve its properties. The nanoparticles used in nanolubricants can be of various materials, such as metal oxides, carbon-based materials, and layered materials. Adding nanoparticles to the base lubricant enhances its properties, such as viscosity, thermal stability, and tribological properties, and helps reduce friction and wear in engine components^2,3^.

Molybdenum disulfide (MoS_2_) is a two-dimensional material with unique tribological properties, making it a promising candidate for lubrication applications. The layered structure of MoS_2_ is characterized by strong interlayer covalent bonds and weak van der Waals forces between molecular layers, allowing the formation of a slippery surface that reduces friction and wear between moving parts^4,5^. The van der Waals gap plays a critical role in intercalation and lubrication properties. The MoS_2_ is used extensively in solid lubrication, lubrication additives in oils, and self-lubricating polymer materials. Various forms of MoS_2_, such as flower-like microspheres and ultrathin nanosheets, have been synthesized and evaluated for their tribological properties. Tang et al. synthesized MoS_2_ flower-like microspheres, which demonstrated superior anti-wear and friction-reducing properties when used as a lubrication additive compared to pure base oil^6^. Song et al. prepared ultrathin MoS_2_ nanosheets as lubricating additives and found that their tribological and extreme pressure properties were significantly enhanced due to the good dispersion and ultrathin shape of the nanosheets^7^. MoS_2_’s unique structural and mechanical properties enable it to reduce friction and wear and prevent deposition, making it an important additive in various industrial applications. Ongoing research is focused on developing innovative techniques to synthesize and utilize MoS_2_ to enhance its tribological properties further and broaden its range of applications. For example, Song et al. synthesized MoS_2_/graphene oxide hybrid nanosheets and reported that they exhibited excellent tribological performance and could be potentially used as lubricant additives^8^. Moreover, Chen et al. synthesized a composite material of MoS_2_ and boron nitride, which possessed superior tribological properties as a lubricating additive^9^.

Graphene, a two-dimensional material of carbon atoms arranged in a hexagonal lattice, possesses exceptional tribological properties that make it an attractive candidate for various industrial applications. Its high chemical inertness, extreme strength, and easy shear capability on its densely packed and atomically smooth surface are favourable attributes for its impressive friction and wear behavior. Numerous recent studies have explored graphene’s potential as a solid or colloidal liquid lubricant. Liang et al.^10^ demonstrated that in-situ exfoliated graphene exhibits exceptional friction-reducing and anti-wear properties. Huang et al.^11^ reported that crumpled graphene balls could significantly improve the lubrication properties of polyalphaolefin (PAO) base oil as a high-performance additive, with tribological performance that surpasses that of other carbon additives, such as graphite and carbon black. The tribological behavior of graphene is also influenced by its surface functionalization, which can be tailored to enhance its lubrication properties. For instance, Tao Bai et al. synthesized polyurethane-graphene oxide and Fe_3_O_4_ nanocomposites and observed a significant reduction in the coefficient of friction and wear rate compared to neat polyurethane^12^.

Molybdenum disulfide (MoS_2_) and graphene have attracted significant attention in the field of tribology due to their unique mechanical and structural properties. MoS_2_, with its layered and two-dimensional structure, exhibits excellent tribological behavior as a solid lubricant due to its low friction and wear properties. On the other hand, graphene possesses exceptional mechanical strength, chemical stability, and easy shear capability on its densely packed and atomically smooth surface. Combining these two materials as a nanocomposite further enhances their tribological properties, making them a valuable additive in various industrial applications. The high shear strength of graphene and the layered structure of MoS_2_ may result in a synergistic effect, reducing friction and wear. Due to their excellent mechanical and structural properties MoS_2_/graphene nanocomposites may emerge as promising materials for tribological applications.

In this research, we used an innovative approach to synthesize MoS_2_-graphene nanohybrid, employing a microwave synthesis platform. This technique has demonstrated a remarkable reduction in both synthesis time and energy consumption by approximately 90–98% compared to conventional synthesis techniques such as solvothermal, chemical vapor deposition (CVD), laser ablation, and others, as previously documented ^13–16^. The primary objective of this study is to investigate and elucidate the underlying mechanisms responsible for the substantial performance enhancement of base oil upon incorporating MoS_2_-graphene nanohybrid additives. In pursuit of this objective, we have conducted a comprehensive examination of several key parameters, including the coefficient of friction (COF), average wear scar diameter (WSD), oxidation induction time (OIT), and thermal conductivity. Notably, this investigation represents a new effort, as no prior research has systematically explored all these crucial parameters in the context of hybrid nanoparticles. Our foremost aim is to understand the influence exerted by hybrid MoS_2_-graphene nanohybrid additives on the behavior of engine oil. This knowledge holds the potential to facilitate the development of advanced lubricants characterized by superior performance characteristics. The outcomes of this research endeavor are poised to yield valuable insights into enhancing engine oil performance by incorporating MoS_2_-graphene nanohybrid additives, specifically within the domain of diesel-based engine oil applications. Consequently, this research may pave the way for a more sustainable future, marked by a reduced carbon footprint and cost-effective maintenance practices within energy conservation and efficiency.

## Results and discussion

### Physiochemical and morphological characterization of MoS_2_-graphene nanohybrid

**Figure 1:**
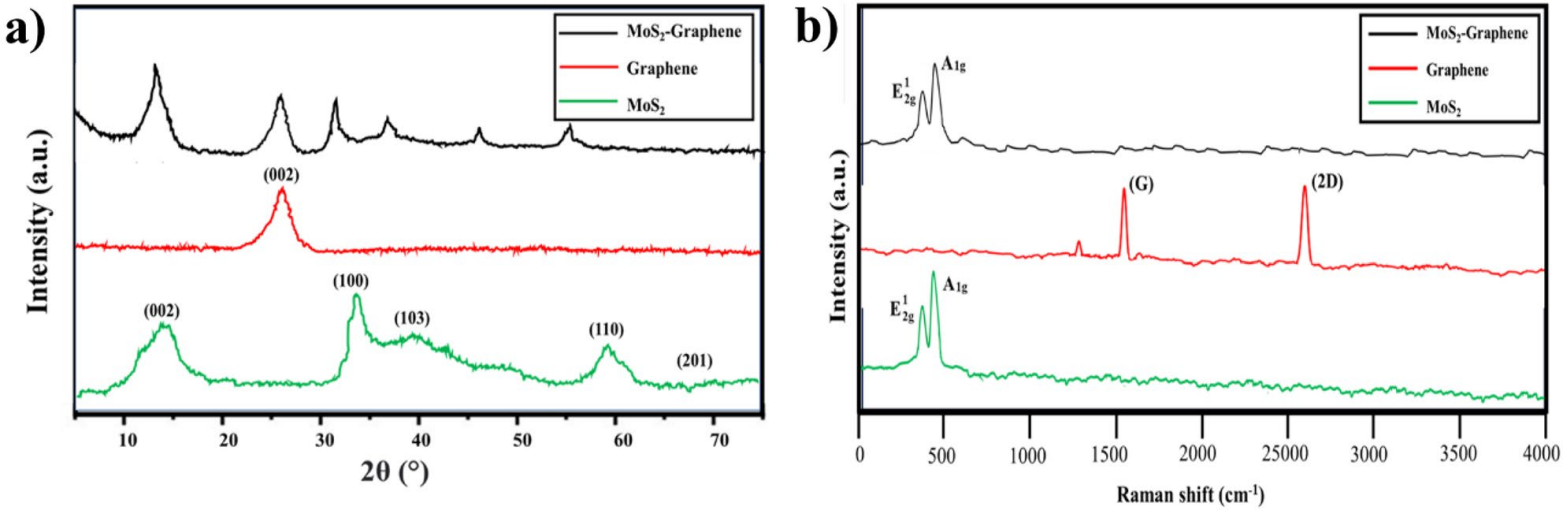
(**a**) XRD spectrum (**b**) Raman spectra of the MoS_2_, Graphene and MoS_2_-Graphene nanohybrid.

Figure 1 shows XRD and Raman spectra analysis of the physiochemical properties of MoS_2_, graphene nanoparticles, and synthesized MoS_2_-graphene nanohybrid. The diffraction peaks at 2θ = 14.5°, 33.0°, 39.3°, 58.5°, and 69.7° in Fig. 1a are connected to the (002), (100), (103), (110), and (201) peaks of the pure MoS_2_ phase (JCPDS no.371492)^17,18^. The graphene diffraction peaks are depicted as a single major peak at 2θ = 24.6° from the (002). The hybrid nanoparticle had no additional peaks or substantial changes from the original MoS_2_ or graphene diffraction spectra. The estimation of crystallite size in the MoS_2_-graphene nanohybrid was conducted using the Scherrer equation:

1 $$D = \frac{K\lambda }{{\beta cos\theta }}$$

Here D represents the crystallite size in nanometers, K is the Scherrer constant with a value of 0.9, λ denotes the wavelength of X-rays, β corresponds to the full width at half maximum (FWHM), and θ represents the peak position. Applying Eq. (1), the calculated crystallite size of the MoS_2_-graphene nanohybrid was determined to be 94.46 nm.

The Raman spectra in Fig. 1b show the respective spectra for MoS_2_, Graphene and MoS_2_-graphene nanohybrid. The Raman spectrum of MoS_2_ shows 2 strong peaks; the first (in 367 cm^−1^) is associated with the E^1^_2g_ vibrational mode, whereas the second (in 406 cm^−1^) is associated with the A_1g_ mode. On the other hand, these modes correspond to in-plane vibrations of sulphur atoms in one direction, molybdenum atoms in the other, and out-of-plane vibrations (A_1g_) of sulphur atoms. The graphene phase also contributes to the Raman spectrum and the characteristic G and 2D peaks of graphene can be observed at around 1580 cm^−1^ and 2700 cm^−1^, respectively. However, it was observed that only MoS_2_ spectra were visible in the Raman spectra of the nanohybrid, while the spectra of graphene were not detected. This phenomenon can be attributed to the strong Raman scattering from MoS_2_ compared to graphene. The electronic band structure of MoS_2_ allows for strong resonance enhancement of the Raman signal. Thus, the Raman scattering from MoS_2_ dominates over the Raman scattering from graphene. Moreover, the laser used in Raman spectroscopy cannot penetrate through the MoS_2_ layers to excite the underlying graphene layers. The MoS_2_ layers encapsulate the graphene layers, so the Raman signal from the graphene layers were not observed^19,20^.

**Figure 2:**
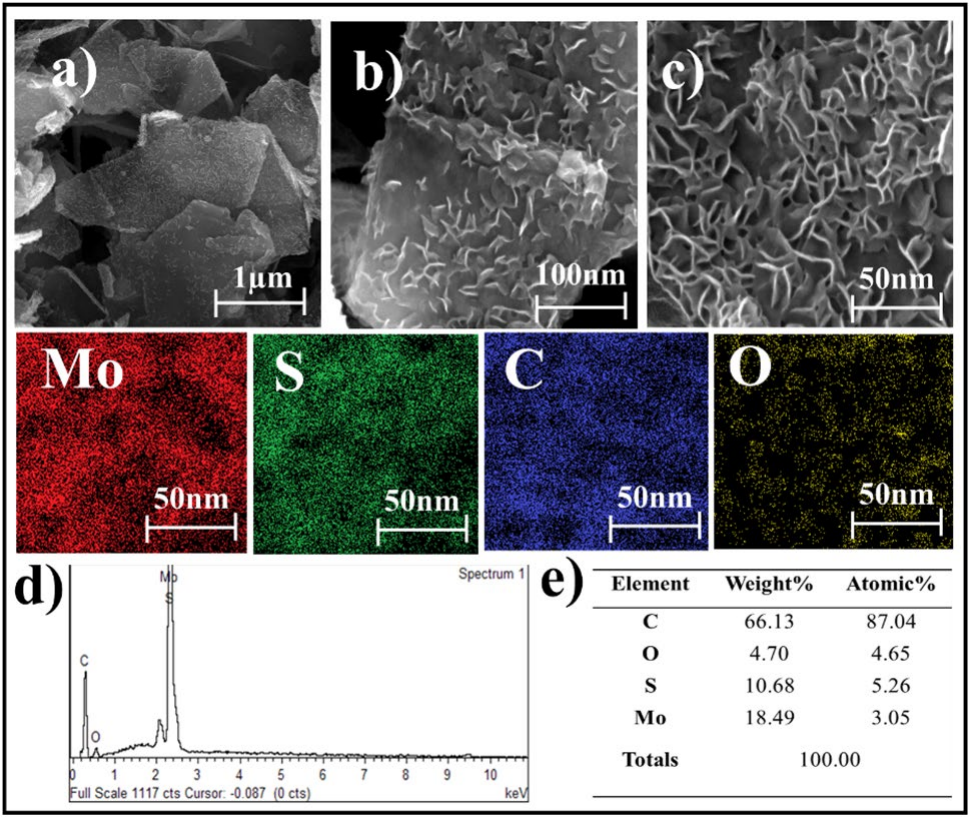
(**a**–**c**) FESEM image at three different magnification levels (Mo), (S), (C) and (O) EDS Mapping composition, (**d**) EDS spectrum, (**e**) elemental distribution of the MoS_2_-graphene nanohybrid.

Figure 2a–c depicts SEM images of MoS_2_-graphene nanohybrid at various magnifications, an EDX spectrum, and elemental mapping. The MoS_2_ nanoparticles are shown to be consistently grown over graphene surfaces with curved edges that are uniformly faceted. The images show regions where the MoS_2_ and graphene are in close proximity, indicating that they are well-dispersed and that there is good interfacial interaction between the two phases. The microwave synthesis platform has proven highly effective in producing a MoS_2_-graphene nanohybrid with intriguing characteristics. Moreover, the resulting hybrid exhibits flake sizes ranging from 100 to 150 nm, confirming the size in the nanometer range. This nano-sized structure imparts significant advantages, primarily promoting efficient reaction kinetics and reducing the likelihood of nanoparticle aggregation^21^. Furthermore, the microwave synthesis method enables the MoS_2_ nanoparticles to encapsulate the surface of graphene sheets and intercalate between the MoS_2_ layers. This unique feature creates an interlayer coupling effect between the hybrid layers, leading to intriguing synergistic properties. The encapsulation of graphene by MoS_2_ and the intercalation between MoS_2_ layers not only enhance the structural integrity of the nanocomposite but also facilitate interfacial interactions and promote interlayer charge transfer processes^22,23^. In Fig. 2, a high-resolution Energy-Dispersive X-ray Spectroscopy (EDS) mapping composition analysis reveals a uniform and homogeneous distribution of Molybdenum (Mo), Sulphur (S), Carbon (C), and Oxygen (O) across the nanosheet of the MoS_2_-graphene nanohybrid. The EDS spectrum in Fig. 2d confirms the presence of Mo, S, C, and O elements within the hybrid MoS_2_-graphene structure. Furthermore, the corresponding quantitative surface analysis depicted in Fig. 2e demonstrates the consistent elemental distribution of these respective elements throughout the hybrid MoS_2_-graphene nanohybrid.

### Physiochemical characterization of MoS_2_-graphene nanolubricant

The findings in Table 1 provide information regarding the density, kinematic viscosity, and viscosity index of a nanolubricant with different concentrations of the MoS_2_-graphene nanohybrid. The results from Table 1 indicate that the addition of the nanohybrid does not lead to any substantial alteration in the density of the engine oil. Based on the data presented in Table 1, it is observed that the addition of the MoS_2_-graphene nanohybrid leads to a slight decrease in the kinematic viscosity of the oil. This reduction in viscosity can be attributed to the lubricating effect of the MoS_2_-graphene nanohybrid, which effectively diminishes the friction between the oil molecules and the moving parts. Consequently, this reduction in frictional resistance facilitates the flow of the oil. The presence of the hybrid MoS_2_-graphene nanohybrid within the oil creates a lubricating boundary layer between the moving parts. This boundary layer acts as a protective barrier, minimizing direct metal-to-metal contact and reducing the energy required for movement. In high shear rate environments, such as those encountered in engine components, the formation of this boundary layer becomes particularly significant^24–26^.

**Table 1:** Density, kinematic viscosity and viscosity index of the nanolubricant with various concentrations of MoS_2_-graphene nanohybrid.

Sample name	Density 40 °C (g/cm^3^)	Kinematic viscosity (mm^2^/s)		Viscosity index
		40 °C	100 °C	
MoS_2_-graphene 0.005 wt%	0.8768	113.59	12.71	104.43
MoS_2_-graphene 0.01 wt%	0.8773	113.64	12.84	106.27
MoS_2_-graphene 0.05 wt%	0.8771	113.52	12.89	107.13
MoS_2_-graphene 0.1 wt%	0.8772	113.71	12.74	104.73
SAE 20W40	0.8770	113.76	12.72	104.53

It can be seen that the MoS_2_-graphene nanohybrid increases the viscosity index in the nanolubricant compared to the SAE20W40. The viscosity index measures the lubricant’s resistance to changes in viscosity with temperature changes. A higher viscosity index indicates that the lubricant will maintain its viscosity over a wider range of temperatures, which is crucial for engine protection and fuel efficiency^27^. The increase in the viscosity index reduces the tendency of the lubricant to thicken at low temperatures and thin at high temperatures. This improves the stability of the lubricant and ensures it provides effective engine protection even under extreme operating conditions^28^.

**Figure 3:**
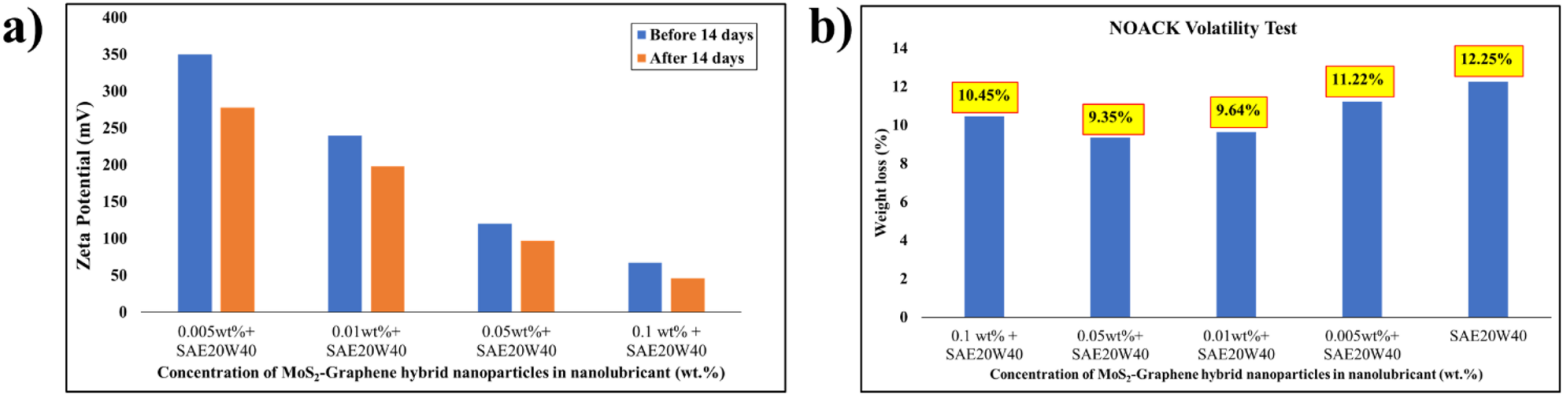
(**a**) The zeta potential analysis, (**b**) the NOACK Volatility analysis of nanolubricant with various concentrations of MoS_2_-graphene nanohybrid.

A zeta potential analysis is conducted to assess the stability of the MoS_2_-graphene nanohybrid-based nanolubricant in engine oil. This analysis is crucial as it measures the electrical charge of the dispersed nanoparticles within the engine oil, providing valuable insights into the stability of the nanoparticles. The measurement of zeta potential is a widely adopted method employed by researchers for studying nanolubricant stability ^29,30^. Figure 3a illustrates the zeta potential values of the nanolubricant containing various concentrations of the MoS_2_-graphene nanohybrid before and after a period of 14 days. The zeta potential values indicate the dispersion intensity of the nanoparticles in the engine oil, directly influencing the stability of the nanolubricant. Based on Fig. 3a, it can be observed that the majority of the nanolubricants investigated in this study exhibited zeta potential values exceeding 60 mV after 14 days of synthesis. These high zeta potential values indicate excellent stability of the MoS_2_-graphene nanohybrid in the engine oil. The increase in zeta potential values is due to the graphene in the nanocomposite having a high surface area and electrical conductivity, which provides a large number of active sites for surface interaction and electrostatic stabilization. The MoS_2_ in the nanocomposite has a high surface energy and a polar surface, which can further enhance the surface interaction and electrostatic stabilization of the oil droplets^31^. This improves the charge distribution at the surface of the oil droplets, leading to higher zeta potential. The high zeta potential may prevent the droplets from agglomerating and sticking to engine components, which improves engine performance and prolongs engine life^32^. The increase in stability reduces the interfacial tension between the oil droplets and the engine components. This may improve the wetting ability of the oil, leading to better lubrication and reduced wear and tear.

The NOACK volatility test ASTM D5800 is a standard measure of the evaporation loss of lubricating oils under high-temperature conditions. In the NOACK volatility test, a nanolubricant sample is heated at 250 °C for 60 min with continuous airflow. The weight loss during this process evaluates the oil’s volatility. The test simulates the engine operation conditions, where high temperatures can cause oil to evaporate and lead to oil thickening, deposits, and reduced engine performance. A lower mass loss in the NOACK test indicates that the oil is less prone to evaporation, which is desirable for engine protection and fuel efficiency. The Noack volatility study of nanolubricant with varying concentrations of MoS_2_-graphene nanohybrid is shown in Fig. 3b. The SAE20W40 evaporative loss was 12.25%, whereas the MoS_2_-graphene nanohybrid based nanolubricants evaporative loss ranged from 9.35 to 11.22%. This can be attributed to several mechanisms of the nanohybrid, which act as a heat sink, absorbing and dissipating the heat generated during engine operation, thereby reducing the evaporation of the lubricant. Furthermore, the nanohybrid reduces the vapor pressure of the nanolubricant engine oil^33^. The vapor pressure is a measure of the tendency of a fluid to evaporate. The MoS_2_-Graphene nanohybrid reduces the vapor pressure of the nanolubricant engine oil by adsorbing onto the metal surfaces and forming a protective layer that reduces the surface area available for evaporation. This can lead to a lower mass loss in the NOACK analysis and provides better engine protection^34^.

### Tribological analysis of MoS_2_-graphene nanohybrid nanolubricant

**Figure 4:**
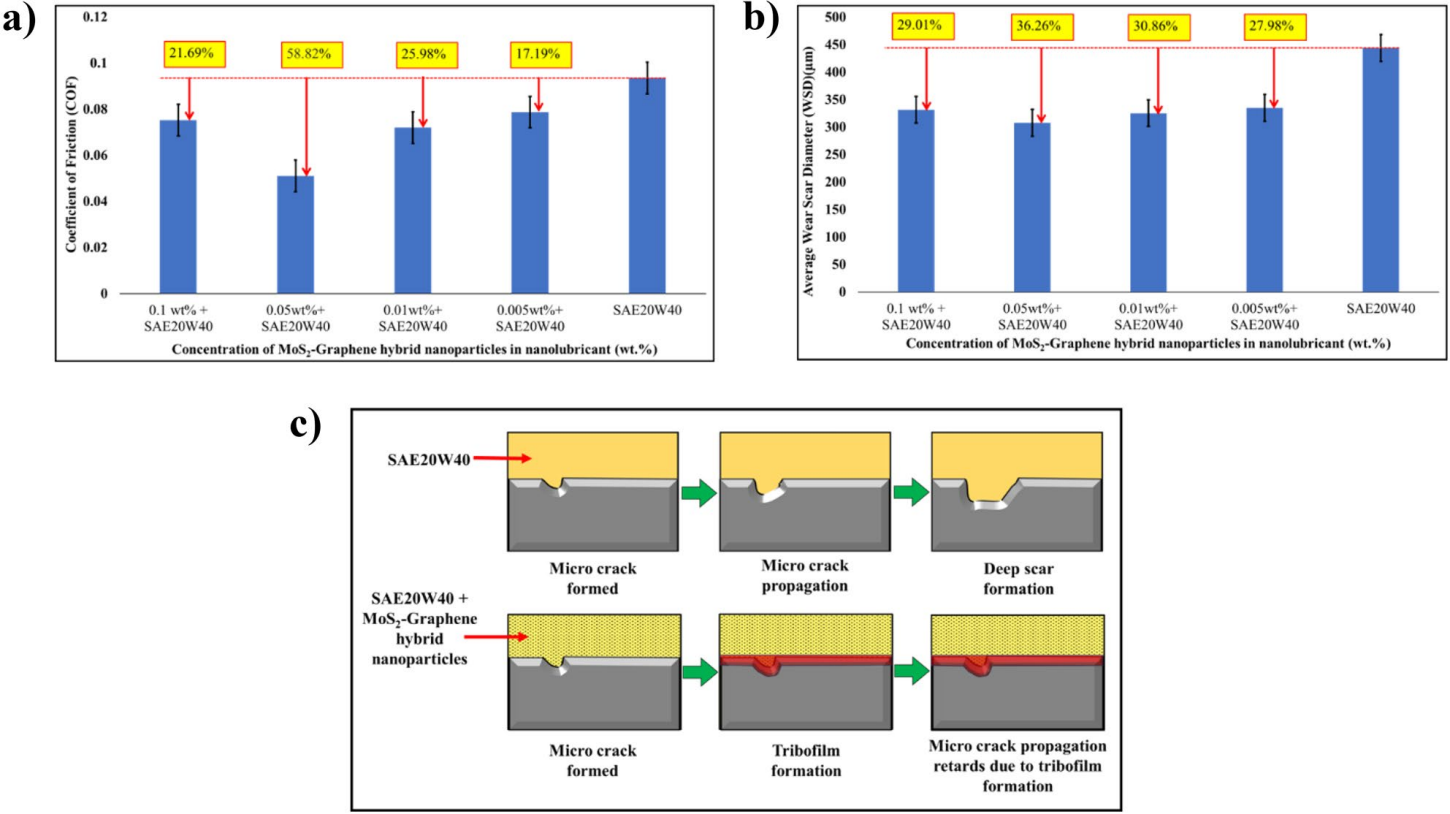
(**a**) The coefficient of friction, (**b**) the average wear scar diameter of MoS_2_-graphene hybrid nanolubricant, (**c**) shematic diagram of lubriacation mechanism of MoS_2_-graphene hybrid nanolubricant.

Figure 4a presents the coefficient of friction (COF) values obtained for the nanolubricant containing different concentrations of the MoS_2_-graphene nanohybrid in engine oil. The COF represents the frictional resistance experienced during lubricated sliding. Upon examination of Fig. 4a, it is evident that the COF of the pure SAE20W40 base oil was measured at 0.0946. When incorporating the MoS_2_-graphene nanohybrid into the base oil, significant reductions in COF are observed. The COF values for the nanolubricant with 0.1 wt%, 0.05 wt%, 0.01 wt%, and 0.005 wt% concentrations of the MoS_2_-graphene nanohybrid were determined to be 21.69%, 58.82%, 25.98%, and 17.19% lower, respectively, compared to the base oil. The reduction in COF is due to a highly effective boundary lubrication mechanism. Boundary lubrication occurs when the lubricant film is in direct contact with the solid surfaces, and the lubricant molecules form a protective layer that prevents metal-to-metal contact. The MoS_2_-graphene nanohybrid is an excellent boundary lubricant because of its unique properties. Firstly, MoS_2_ and graphene nanoparticles have a layered structure with weak van der Waals forces between the layers. This allows the nanoparticles to easily slide over each other, creating a low-friction surface. Secondly, graphene has a high surface area and may adsorb onto metal surfaces, providing additional lubrication and protection. When MoS_2_-graphene nanohybrid is added to nanolubricant engine oil, it forms a self-assembled layer on the metal surfaces due to its high affinity to metals^35,36^. This layer acts as a barrier between the surfaces and reduces friction by preventing metal-to-metal contact. The MoS_2_-graphene nanohybrid can also withstand high pressures and temperatures, commonly found in engine operation, without degrading or losing its lubricating properties. Additionally, the MoS_2_-graphene nanohybrid as an additive in the engine oil also reduces wear and tear on the engine parts, leading to longer engine life and improved performance. This is because the MoS_2_-graphene nanohybrid forms a protective tribofilm on the metal surfaces, which acts as a sacrificial layer that absorbs the wear and tear, thereby reducing the damage to the underlying metal surfaces^7,37^. When the nanohybrid concentration exceeds 0.05 wt%, the COF rises owing to nanoparticle aggregation. When nanoparticles cluster together, they can generate hard particles that behave as abrasives, increasing surface friction.

The average wear scar diameters (WSD) MoS_2_-graphene nanohybrid in SAE20W40 diesel engine oil were measured using an optical profilometer in Fig. 4b. Without the inclusion of nanoparticles, the WSD of base oil was 444 µm with the addition of 0.05 wt% MoS_2_-graphene, the WSD of the nanolubricant was lowered by up to 36.26%. The MoS_2_-graphene nanohybrid is a highly effective lubricant additive that reduces the average wear scar diameter in nanolubricant engine oil. The MoS_2_-graphene nanohybrid is a highly effective lubricant additive that may reduce wear by several mechanisms. Firstly, the MoS_2_-graphene nanohybrid forms a self-assembled layer on the metal surfaces, which acts as a protective barrier that absorbs wear and tear. This layer reduces the amount of material that is removed from the metal surfaces, leading to a lower wear scar diameter. Secondly, the MoS_2_-graphene nanohybrid reduces wear by reducing friction between the metal surfaces. As discussed earlier, the MoS_2_-graphene nanohybrid is a highly effective boundary lubricant that reduces friction by forming a low-friction surface on metal surfaces^38^. This reduces the amount of mechanical wear that occurs during engine operation. Thirdly, the MoS_2_-graphene nanohybrid reduces wear by improving the thermal and chemical stability of the lubricant. The MoS_2_-graphene nanohybrid is highly resistant to thermal and chemical degradation, which may prolong the life of the lubricant and reduce the amount of wear that occurs due to thermal and chemical processes^39^. Figure 4c shows the schematic diagram of the lubrication mechanism of MoS_2_-graphene nanolubricant.

**Figure 5:**
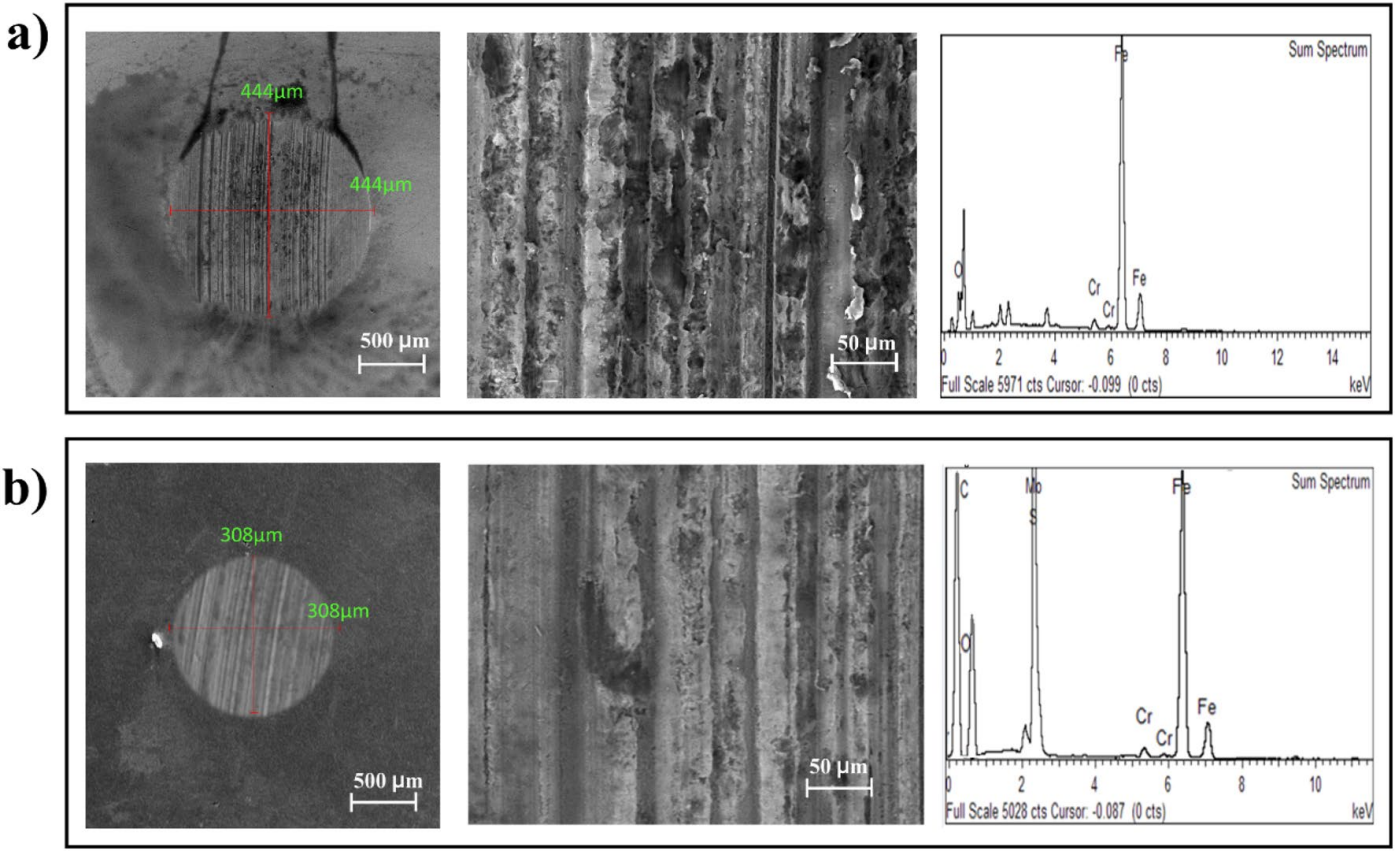
FESEM image of wear scar and corresponding EDX elemental spectrum of (**a**) of base oil without additive, (**b**) base oil with 0.05 wt% hybrid MoS_2_-graphene nanohybrid.

The wear scars on a ball bearing surface resulting from tribological testing were examined using FESEM, as shown in Fig. 5a,b. The FESEM images provided insights into the wear surfaces, allowing for a comparison between the wear scar formed on the ball bearing surface lubricated with base oil and the wear scar formed on the ball bearing surface lubricated with a nanolubricant containing 0.05 wt% hybrid MoS_2_-graphene nanohybrid. Figure 5a,b displays the FESEM image of the wear scar, highlighting a substantial difference between the two scenarios. The wear scar observed on the steel ball bearing surface lubricated solely with the base oil exhibited a significantly deeper and more pronounced scar compared to the wear scar formed on the ball bearing surface lubricated with the nanolubricant containing 0.05 wt% hybrid MoS_2_-graphene nanohybrid. This disparity suggests that the addition of nanoparticles, specifically the hybrid MoS_2_-graphene nanohybrid, exerted a mending effect on the wear surface^5^. The nanoparticles were hypothesized to deposit within the micro-cracks and defects present on the mating surfaces, thereby facilitating the repair and smoothening of the surface. This mending effect results in a smoother and more uniform surface, reducing the wear scar depth ^40^. The presence of the hybrid MoS_2_-graphene nanohybrid was confirmed by examining the Elemental Dispersive X-ray (EDX) spectra. The EDX elemental spectrum revealed the detection of Mo, S, C, and O elements on the wear scar of the ball bearing lubricated with the nanolubricant containing 0.05 wt% hybrid MoS_2_-graphene nanohybrid. Conversely, no detection of the nanoparticle elements was observed on the wear scar of the ball bearing lubricated solely with the base oil (Fig. 5a).

### Oxidation induction time and thermal conductivity analysis of MoS_2_-graphene nanolubricant

**Figure 6:**
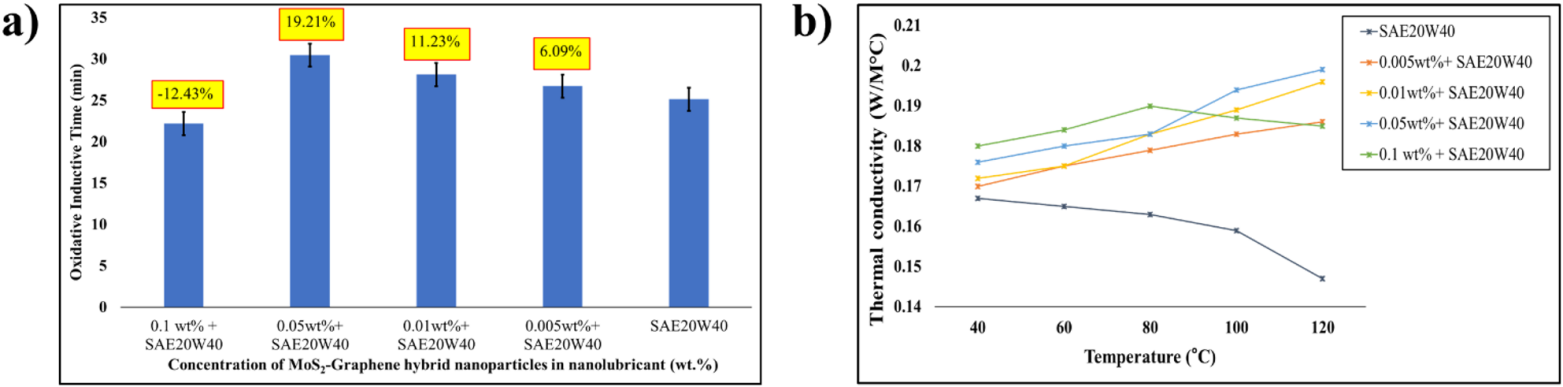
(**a**) OIT analysis and (**b**) thermal conductivity of hybrid MoS_2_-graphene nanolubricant.

The oxidative induction time (OIT) measures the resistance of the lubricant to oxidation, which leads to the formation of deposits and sludge in the engine, reduced lubricant performance, and increased engine wear. Figure 6a depicts the OIT of a nanolubricant in engine oil with different concentrations of MoS_2_-graphene anohybrid. With the addition of 0.05 wt% of the nanohybrid, the OIT of the nanolubricant increased to 19.21%. The MoS_2_-graphene nanohybrid improved oxidative stability by several mechanisms. Initially, the nanohybrid neutralizes the reactive species generated during oxidation reactions. This inhibits oxidation reactions by forming a protective barrier that prevents oxygen from reaching the lubricant components^41^. Subsequently, the MoS_2_-graphene nanohybrid improves oxidative stability by reducing the metal catalyst effect. Metal catalysts such as copper, iron, and nickel accelerate oxidation reactions by promoting the formation of reactive species. The MoS_2_-graphene nanohybrid may form a self-assembled layer on the metal surfaces, which acts as a barrier that reduces the metal catalyst effect and slows down oxidation reactions^42,43^. Lastly, the nanohybrid improves oxidative stability by reducing deposits and sludge formation. It acts as a dispersant, preventing the accumulation of deposits and sludge by keeping the lubricant components suspended. When the nanohybrid concentration exceeds 0.05 wt%, the OIT deteriorates due to the aggregated nanoparticles that created localized hotspots within the oil, leading to increased oxidative reactions and decreased OIT. Aggregated particles may also hinder the access of antioxidant molecules to the oil, further reducing the oxidative stability of the nanolubricant.

The thermal conductivity of a lubricant plays a vital role in the efficiency of engine operation. In general, higher thermal conductivity leads to better heat dissipation, which can reduce the operating temperature of engine components, increase fuel efficiency, and prolong engine life. Figure 6b indicates that incorporating MoS_2_-graphene nanohybrid at various concentrations into SAE20W40-based diesel oil considerably enhanced engine oil thermal conductivity. In the present study, the incorporation of 0.05 wt% MoS_2_-graphene nanohybrid has been demonstrated to significantly enhance heat conductivity by 19.83% (at 100 °C). This enhancement can be attributed to the formation of a thin, continuous film of nanocomposite on the metal surfaces of engine components. The presence of this film promotes better thermal contact between the components and the lubricant, thereby facilitating efficient heat transfer from the engine components to the lubricant. Additionally, the MoS_2_-graphene nanohybrid contributes to the improved dispersion of the lubricant, resulting in enhanced heat transfer throughout the lubricant system. Consequently, utilizing this nanohybrid exhibits the potential to dissipate heat and improve overall thermal performance effectively.

The nanohybrid facilitates the formation of a network-like structure in the lubricant, known as percolation mechanism, which can increase the thermal conductivity of the oil. The MoS_2_ in the nanohybrid acts as a bridge between the graphene layers, enhancing the thermal conductivity of the composite^44,45^. The thermal conductivity decreases drastically at 0.1 wt% nanohybrid concentration. The higher nanohybrid concentration causes it to aggregate, forming an insulating surface layer in the nanolubricant. These layers can act as thermal barriers, limiting heat transfer between the oil molecules and the nanoparticles, thus reducing the overall thermal conductivity^46^.

## Conclusion

In conclusion, this research study successfully investigated the tribological behavior of hybrid MoS_2_-graphene nanohybrid in SAE20W40 diesel-based engine oil. The utilization of the advanced microwave synthesis platform proved to be highly efficient, significantly reducing synthesis time and energy consumption. The synthesis process resulted in the uniform growth of MoS_2_ nanoparticles on the surface of Graphene nanoparticles through the interaction between their respective functional groups. The addition of 0.05 wt% hybrid MoS_2_-graphene nanohybrid in SAE20W40 diesel-based engine oil demonstrated remarkable improvements in various performance aspects. Specifically, a significant reduction of 58.82% in the coefficient of friction and 36.26% in the average wear scar diameter was observed compared to the base oil. Moreover, the nanolubricant exhibited notable enhancements in oxidation analysis by 19.21% and thermal conductivity by 19.83% at 100 °C. These findings confirm that the microwave synthesis of hybrid MoS_2_-graphene nanohybrid offers excellent potential as tribological additives in diesel-based engine oil. The synthesized nanoparticles significantly improve tribological performance, oxidation resistance, and thermal conductivity when incorporated into the engine oil. This research provides valuable recommendations for future studies to advance the application of hybrid MoS_2_-graphene nanohybrid. Key focal points include refining surface functionalization techniques to enhance compatibility with engine oil, conducting rigorous environmental impact assessments, and validating compatibility through thorough engine testing. Additionally, efforts should concentrate on assessing the scalability of nanoparticle synthesis for commercial production and exploring diverse industrial applications. These areas of emphasis are pivotal for realizing the full potential of hybrid MoS_2_-graphene nanohybrid in diverse applications. These advancements can potentially enhance the durability and efficiency of mechanical components in engine systems. The synergistic effects of the nanohybrid provide a promising avenue for future research and development of advanced lubricant formulations.

## Experimental details

### Materials

For the experimentation, all reagents used in synthesizing hybrid MoS_2_-graphene nanohybrid were of analytical grade and employed without further purification. Ammonium molybdate tetrahydrate ((NH_4_)6Mo_7_O_24_.4H_2_O) was procured from Fisher Chemicals located in Chicago, USA, whereas thiourea (SC(NH_2_)_2_) was sourced from R&M Chemicals based in Dundee, UK. Graphene with a thickness of 8 nm and a specific surface area of approximately 80m^2^/g was obtained from Graphene Labs Inc, USA. The lubricant oil employed in the experimentation was diesel engine oil with API SAE20W40 CD/SE GL-4.The properties and specifications of the engine oil is shown in Table 2.

**Table 2:** The properties and specifications of SAE20W40 diesel based engine oil used in this experiment.

Property	Specification
Grade	SAE 20W-40
Pour Point, °C, ASTM D97	− 24
Flash Point, °C, ASTM D92	233
Density @ 15 °C, g/mL, ASTM D1298	0.879
Total base number, mgKOH/g, ASTM D2896	6
Kinematic Viscosity @ 100 °C, mm^2^/s, ASTM D445	12.72
Kinematic viscosity @ 40 °C, mm^2^/s, ASTM D445	113.76
Ash, sulfated, mass %, ASTM D874	0.08

### Synthesis of hybrid MoS_2_-graphene nanohybrid

**Figure 7:**
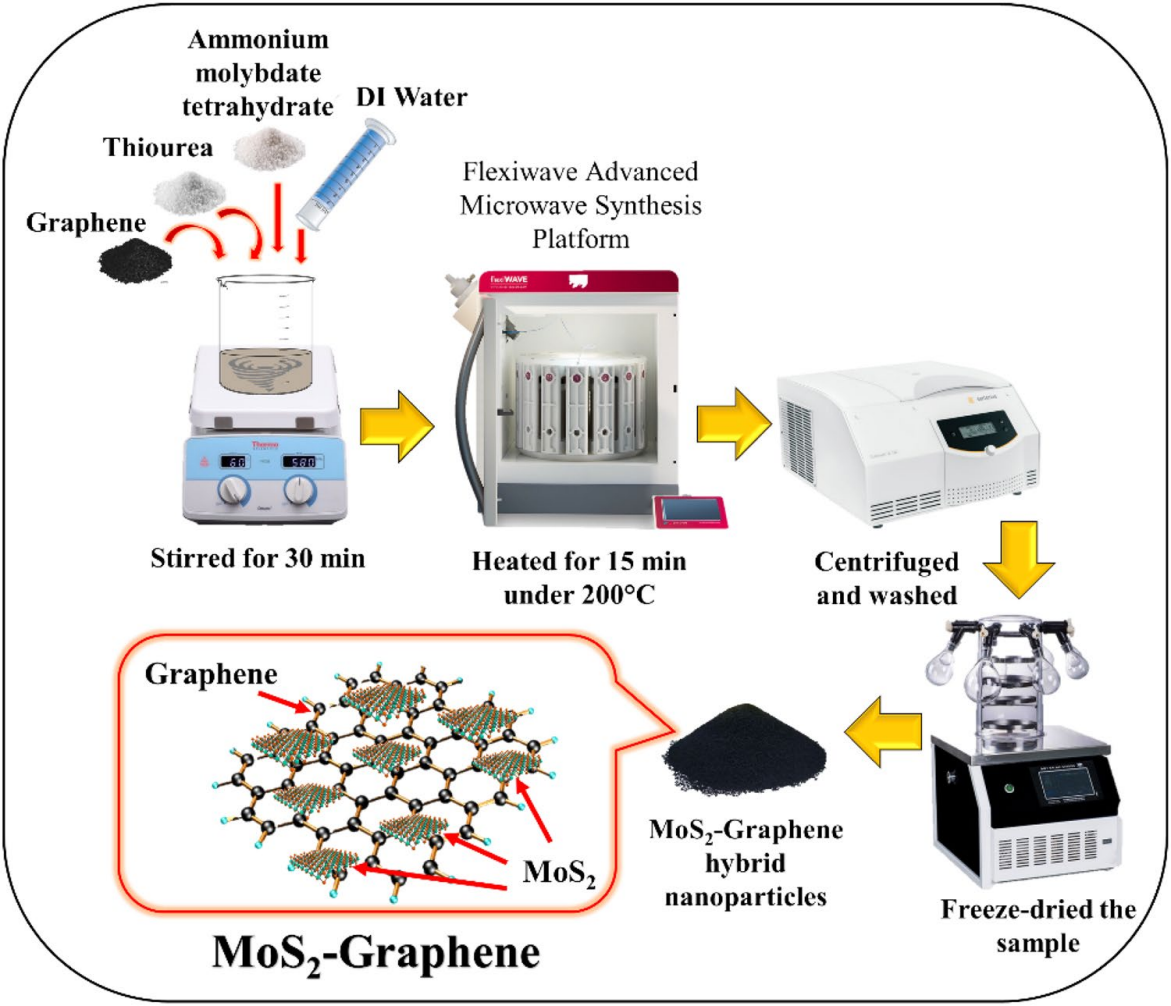
The schematic illustration of synthesizing method of hybrid MoS_2_-graphene nanohybrid.

The researchers utilized a microwave hydrothermal synthesis technique to synthesize a hybrid MoS_2_-graphene nanohybrid. In this experimental approach, a solution was prepared by gradually adding 3.7 g of ammonium molybdate tetrahydrate and 6.85 g of thiourea to 105 mL of deionized water. The mixture was stirred for 30 min to ensure homogeneity. Subsequently, 1 g of graphene powder was introduced, and the resulting mixture was subjected to sonication for 30 min. The solution was then transferred to a microwave synthesis platform (Milestone, flexiWAVE, Italy) and heated to 200 °C for 15 min. After heating, the sample was allowed to cool down to room temperature (26 °C) naturally. Subsequently, the sample was centrifugated, washed with distilled water and ethanol, and finally freeze-dried. The synthesis method of the hybrid MoS_2_-graphene nanohybrid is illustrated in Fig. 7.

### The hybrid MoS_2_-Graphene nanolubricant formulation

Various concentrations of hybrid MoS_2_-graphene nanohybrid, including 0.005 wt%, 0.01 wt%, 0.05 wt%, and 0.1 wt%, were dispersed in 100 ml of SAE 20W40 engine oil. The mixture was then homogenized for 10 min using a high-shear lab mixer. To ensure that the nanoparticles were uniformly dispersed in the base oil and to prevent agglomeration, the samples were sonicated in an ultrasonic bath for 30 min.

### Characterizations

This study utilized various analytical and characterization methods to investigate the properties and performance of hybrid MoS_2_-graphene nanohybrid as a lubricant additive. The size, morphology, and elemental compositions of the nanohybrid were determined using field emission scanning electron microscopy and energy-dispersive X-ray spectroscopy (FESEM and EDX). X-ray diffraction (XRD) with Cu Kα radiation (U = 45 kV, I = 27 mA, and λ = 1.54 nm) was employed to study the crystallinity and phase structure of the samples, while Raman spectroscopy was used to analyze the molecular interactions using a 532 nm laser. Additionally, the density, kinematic viscosity, and viscosity index of the samples were measured with a viscometer (Viscometer SWM 3000), and Zetasizer was used to determine the dispersion stability. The Noack volatility test was performed using Thermogravimetric Analysis (TGA). In the NOACK volatility test, nanolubricant engine oil is heated at 250 °C for 60 min with constant airflow. The resulting weight loss indicates the oil’s volatility characteristics, offering insights into its performance under high-temperature conditions.

Tribological analysis was conducted using a four-ball tribotester (Ducom TR-30L) to measure the coefficient of friction (COF) and average wear scar diameter (WSD) of the hybrid MoS_2-_Graphene based nanolubricant at various nanoparticle concentrations. Carbon chromium steel balls (Hardness (H), HRC: 65, Density (ρ), gm/cm: 37.79 Surface roughness (Ra), μm: 0.022) were employed, and the rotational speed, applied load, time, and temperature were set to 12,000 rpm, 392.5 N, 3600 s, and 75 °C, respectively, in accordance with ASTM 4172-94. The wear scar images on the metal balls were studied using FESEM and EDX.

Furthermore, oxidation analysis was performed using High-Pressure Differential Scanning Calorimeter (HP-DSC) to determine the oxidation induction time (OIT) of MoS_2_-graphene based nanolubricant with various nanohybrid concentrations. The procedure was carried out at 500 psi, an isothermal temperature of 200 °C, a flow rate of 50 ml/min, and ramping rate of 10 °C/min. The thermal conductivity of the MoS_2-_graphene based nanolubricant with different nanohybrid concentrations was evaluated using laser flash analysis (LFA HyperFlash). The sample was filled in the sample ring, and the upper and lower sealing discs were sprayed with graphite to promote black body absorption. The sample was heated from room temperature to 120 °C at a rate of 10 °C/min in a nitrogen environment.

## Data Availability

The datasets analyzed in the current study are available from the corresponding author upon reasonable request.
